# Matairesinol exerts anti-inflammatory and antioxidant effects in sepsis-mediated brain injury by repressing the MAPK and NF-κB pathways through up-regulating AMPK

**DOI:** 10.18632/aging.203649

**Published:** 2021-10-27

**Authors:** Qin Wu, Yuhua Wang, Qingfang Li

**Affiliations:** 1 Rehabilitation Medicine Department, Shanxi Provincial People’s Hospital, Taiyuan 030001, Shanxi, China

**Keywords:** Matairesinol, sepsis, brain injury, neuroinflammation, microglia, AMPK

## Abstract

Brain injury is a familiar complication of severe sepsis, in which excessive inflammation and oxidative stress are the main mechanisms leading to acute brain injury. Here, we focus on probing the function and mechanism of Matairesinol (Mat) in sepsis-mediated brain injury. We established a rat sepsis model by cecal ligation and perforation (CLP) and constructed an *in vitro* sepsis model by treating neurons and microglia with lipopolysaccharide (LPS). Rats and cells were treated with varying concentrations of Mat, and the changes of neural function, neuronal apoptosis, microglial activation, neuroinflammation and the expression of oxidative stress factors in brain tissues were examined. Additionally, the activation of the MAPK, NF-κB and AMPK pathways in brain tissues and cells was evaluated by Western blot (WB) and/or immunohistochemistry (IHC). Our findings illustrated that Mat improved neuronal apoptosis and weakened microglial activation in CLP rats. Meanwhile, Mat hampered the expression of pro-inflammatory factors (TNF-α, IL-1β, IL-6, IFN-γ, IL-8, and MCP1) and facilitated the contents of glutathione peroxidase (GSH-Px) and superoxide dismutase (SOD) in brain tissues and microglia. Mechanistically, Mat concentration-dependently dampened the phosphorylation of MAPK, JNK and NF-κB in CLP rats and LPS-stimulated microglia and up-regulated Nrf2 and HO-1. Besides, Mat facilitated the AMPK expression. Meanwhile, Compound C, a specific inhibitor of the AMPK pathway, substantially reduced the neuronal protection and anti-inflammatory effects mediated by Mat. Overall, Mat exerts anti-inflammatory and anti-oxidative stress effects by up-regulating AMPK, thereby ameliorating sepsis-mediated brain injury.

## INTRODUCTION

Sepsis is a frequent infectious disease that was redefined as organ dysfunction infection in 2016 [1]. Sepsis is among the prime contributor to death in critically ill patients, affecting approximately more than 30 million people every year globally, according to a WHO report in 2018 [2]. Sepsis causes dysfunction of multiple organs, with the brain being the first to be affected [3]. Cerebral injury caused by sepsis is an acute complication, which in turn leads to sepsis deterioration and high mortality [4]. Therefore, it is necessary to explore new therapeutic approaches to deal with sepsis-induced brain damage.

Microglia are important players in the homeostasis of the mammalian central nervous system, and their dysregulation causes neurodegenerative and neuroinflammatory diseases [5]. Studies have stated that microglia affect the function of the central nervous system by driving synaptogenesis, synaptic pruning, neurogenesis, and neuronal activity [6]. When external stimulation causes neuronal injury, microglia are spurred to secrete various inflammatory factors. Excessive activation of microglia brings about damage to the healthy peripheral neural tissue, while factors secreted by dead or dying neurons in turn intensify the long-term activation of microglia, resulting in progressive neuronal loss [7]. Oxidative stress accompanying sepsis is a major facilitator and regulator of the systemic inflammatory response. Oxidative stress destabilizes hemodynamics and leads to multi-organ failure due to systemic inflammatory syndrome [8]. Based on the characteristics of sepsis, several therapeutic drugs with anti-inflammatory or antioxidant effects have been developed. For example, some reports have stated that resveratrol improves sepsis-induced organ damage, prolongs the survival time, and reduces the mortality of septic animals through anti-inflammatory, anti-infectious, antioxidant and functions and by facilitating microcirculation [9]. Therefore, we explored whether drugs with anti-inflammatory or antioxidant effects have significant efficacy in sepsis.

Lignans are a set of natural phenolics found in around 70 plant families, such as in trees, grasses, grains, and vegetables. They have plentiful biological activities such as antibacterial and insecticidal effects in plants and tumor-suppressive, antiviral, anti-inflammatory, immunosuppressive, anti-diabetic, and antioxidant properties in mammals. They have attracted increasing attention due to their potential anti-tumor, antioxidant and other activities [10]. Matairesinol (Mat) is a dibenzyl butyl lactone plant lignan, which has antiangiogenic, anti-inflammatory, antioxidant, anti-tumor and other biological activities. It has been reported that Mat exhibits significant anti-proliferative and pro-apoptotic activities on T-cell lymphoma CCRF-CEM cells, represses cell cycle progression, and enhances the level of reactive oxygen species (ROS) [11]. Other studies have displayed that Mat, a small natural molecule identified from a cellular screen of 200 natural plants, abates the generation of mitochondrial ROS (mROS), thus exhibiting antiangiogenic activities. Besides, Mat represses the hypoxia-inducible factor-1α in hypoxia HeLa cells [12]. Moreover, Mat shows an effective inhibitory activity against NO production in lipopolysaccharide (LPS)-activated SD rat macrophages [13]. However, the role and potential mechanisms of Mat in sepsis have not been evaluated.

The regulatory function of the mitogen-activated protein kinase (MAPK) and nuclear factor κB (NF-κB) pathways in sepsis has been confirmed [14]. MAPK signaling cascades are highly conserved among species and trigger cellular self-regulation by transmitting external signals to the nucleus [15]. NF-κB is an essential transcription factor that modulates inflammation and innate immune function. Following the activation of its upstream pathway, such as TLR4, MyD88 [16], TRAF6 [17], and TAK1 [18], NF-κB is phosphorylated and translocated into the nuclear, thus mediating the transcription of multiple inflammatory cytokines, including IL-6, IL-1β, TNF-α, IFN-γ. Those inflammatory cytokines further aggravate organ injuries [19]. In addition to NF-κB, the JAK-STAT signaling pathway is also implicated in sepsis-induced apoptosis and cell cycle arrest by controlling inflammatory reactions. For instance, microRNA-218 hampers the release of inflammatory factors in the serum by suppressing the JAK/STAT pathway and reversely modulating VOPP1, thereby delaying the progression of sepsis in mice [20]. Also, the JAK2/STAT3 pathway-induced miR-181b intensifies blood-brain barrier damage in septic rats by targeting the down-regulation of sphingosine-1-phosphate receptor 1 (S1PR1) [21]. Recent studies have revealed that targeting those inflammatory pathways makes much sense in treating sepsis. For example, a novel DHA-derived lipid mediator, Maresin 1, reduces neutrophil infiltration, inactivates NF-κB/STAT3/MAPK, and regulates the content of inflammatory cytokines to relieve inflammation and reduce sepsis-related acute kidney injury in mice [22]. Besides, the porcine lactoferrin peptide (LFP)-20 prevents LPS-induced inflammation in porcine alveolar macrophages by inactivating MyD88/NF-κB and MyD88/MAPK both *in vitro* and in mouse models [23]. Nevertheless, it is not clear whether the role of Mat in sepsis-mediated brain injury is associated with the MAPK and NF-κB pathways.

Overall, this study investigates the role and related mechanisms of Mat in sepsis-induced brain injury. Through a series of *ex vivo* and *in vivo* tests, we found that Mat significantly improves LPS-mediated neuronal injury and microglial inflammation. Additionally, Mat dampens MAPK and NF-κB pathways and up-regulates AMPK concentration-dependently. Therefore, we hypothesized that Mat regulates sepsis-mediated nerve injury and neuroinflammation through the AMPK pathway.

## MATERIALS AND METHODS

### Cell culture

Neurons (NSC-34 and HT22) and BV2 microglia were acquired from the Cell Center of the Chinese Academy Sciences (Shanghai, China). They were inoculated in the DMEM medium, which comprised 10% FBS and 1% penicillin/streptomycin, and cultured at 37°C with 5% CO_2_. The cell growth was monitored regularly, and the medium was altered every 2–3 days. The cells in the logarithmic growth phase were used for the following experiments.

### MTS assay

The proliferation of NSC-34 and HT22 was examined using an MTS assay kit (Promega Corporation, USA). The optical density was observed with an enzyme-linked immunosorbent assay (ELISA) reader (MD SpectraMax M5; Molecular Devices, LLC, USA) at 492 nm.

### Flow cytometry (FCM)

After treatment with different factors, NSC-34 and HT22 cells were trypsinized and centrifuged (1500 RPM, 3 min). The obtained cells were operated according to the instructions of the apoptosis detection kit (Shanghai Aladdin Bio-Chem Technology Co., LTD). Briefly, the cells were rinsed twice with PBS, followed by the addition of 400 μL pre-cooled PBS. Then, 10 μL AnnexinV-FITC and 5 μL PI were supplemented. After incubation in the dark for 30 min at 4°C, cell apoptosis was gauged by FCM. The apoptotic cell percentage was calculated by a computer software process.

### Western blot (WB)

After NSC-34 and HT22 cells were treated with different factors, the primary culture medium was removed, and the cells were lysed with the RIPA lysate (containing 1% PMSF) and collected via low-speed centrifugation. Then, the total cellular protein was extracted and quantified by the Bradford method. Afterward, the samples underwent boiling for 5 min, cooling on ice and centrifugation for 30 s. The supernatant was harvested for polyacrylamide gel electrophoresis and then transferred to polyvinylidene fluoride (PVDF) membranes at 100 V for one hour. Subsequently, the membranes were sealed with 5% skim milk at room temperature (RT) for one hour and subjected to incubation with the primary antibodies of the Bcl2 (1:1000, ab32124), Caspase-3 (1:1000, ab13847), β-actin (1:1000, ab8226), Nrf2 (1:1000, ab137550) ), HO-1 (1: 2000, ab13243), TBP (1:2000, ab63766), p-MAPK (1:2000, ab170099), MAPK (1: 2000), ab195049), p-JNK (1:1000, ab124956), JNK (1:1000, ab179461), p-NF-κB (1:1000, ab28856), NF-κB (1:1000, ab16502), p-AMPK (1:2000, ab133448), AMPK (1:1000, ab207442), β-actin (1:1000, ab8227), and GAPDH (1:1000, ab9484) at 4°C overnight. Next, the membranes were cleaned twice with TBST and maintained with fluorescein-labeled Goat Anti-Rabbit IgG (1:2500, ab6721) at RT for one hour. After being washed three times, the membranes were exposed with the ECL chromogenic agent and imaged using a membrane scanner. The above antibodies were purchased from Abcam (MA, USA).

### Detection of oxidative stress markers in cells, serum and brain tissues

After microglia were treated with different factors, they were collected and homogenized in 500 μL PBS using ultrasonic cell crushers. Then, they were subjected to centrifugation at 4°C at 1200 RPM for 10 min to obtain the supernatant. After the experiment, 1.5 to 2.5 mL tail blood was extracted from each group and placed in a refrigerator at 4°C overnight. Then, the serum was gathered via centrifugation at 3000 RPM for 10 min. The rats’ brain tissues and normal saline were mixed at a ratio of 1:9. The mixture was homogenized on ice and centrifuged at 420 RPM for 10 min. The BCA protein detection kit (Beyotime) was employed to test the contents of MDA, SOD, CAT and GSH-Px in the cell supernatant and the concentration of MDA and SOD in the serum and brain tissue homogenates according to the regulations.

### ELISA

During the logarithmic growth phase, microglia were seeded into 6-well plates, with four repetitive wells in each group. After further culture for 48 hours, the cell supernatant was harvested and centrifuged at 1000 RPM at 4°C for 10 min. Then, the supernatant was extracted and tested according to the ELISA kit (R&D Systems, Shanghai, China). After the rats’ brain tissues were weighed, the lysate containing protease inhibitors was added for homogenization. Afterward, the homogenate was centrifuged at 14000 RPM at 4°C for 25 min, and the supernatant was retained. The contents of interleukin-1β (IL-1β), interleukin-6 (IL-6), tumor necrosis factor (TNF)-α, interferon (IFN)-γ, interleukin-8 (IL-8), and monocyte chemoattractant protein 1 (MCP1) were gauged by the ELISA kit in cell supernatant and brain tissues, respectively, in strict accordance with the kit requirements.

### The rat sepsis model

This experiment was authorized by the Animal Ethics Committee of Shanxi Provincial People’s Hospital. All of the 30 experimental rats (6–8 weeks old) were bought from the Animal Experimental Center of Wuhan University and raised in the same environment, with free access to food and water.

The rats were anesthetized intraperitoneally (ip) with pentobarbital (30 mg/kg), and cecal ligation and puncture (CLP) was adopted to construct a rat sepsis model. In brief, all rats were fasted for 12 hours before the surgery and were allowed to drink water. After routine surgical disinfection, a 2-cm incision was made along the midline of the abdomen to expose the cecum, which was then ligated at the ileocecal junction without intestinal obstruction. The cecum was punctured once with an 18-gauge needle, and the fecal contents were allowed to penetrate the peritoneum by gently squeezing the cecum. The intestines were then returned to the abdomen, and the abdominal cavity was closed. Rats in the sham group were subjected to laparotomy in which the cecum was manipulated but neither ligated nor punctured. After the surgery, rats in each group were subcutaneously injected with 0.90% sodium chloride solution (30 mL/kg) for resuscitation. To minimize surgical error, all rats were operated on by the same operator. Different concentrations (5, 10, 20 mg/kg) of Mat were administered orally to each group of rats 24 hours after the surgery. Experimental rats were divided into 5 groups: A, sham group; B, CLP group; C, CLP +Mat (5 m/kg) group; D, CLP + Mat (10 mL/kg) group; E, CLP + Mat (20 mL/kg) group. All rats were executed 48 hours after the drug action. Their brain tissues were isolated and preserved at −80°C for subsequent analysis.

### Blood analysis

Rats were anesthetized with ketamine (100 mg/kg, ip) and xylazine (10 mg/kg, ip) mixture, and the blood was taken via cardiac puncture 24 hours following the modeling. Blood samples were centrifuged in a 2 mL Protein LoBind tube (Eppendorf) at 10,000 RPM for 10 min, and then the serum supernatant was transferred to a 0.5 mL Protein LoBind tube (Eppendorf) and stored at −80°C. S100β (EZHS100B-33K, Human S100B ELISA, EMD Millipore), GFAP (NS830, GFAP ELISA, EMD Millipore), and NSE (MBS702407, Mouse NSE ELISA, MyBioSource) in the serum were analyzed using ELISA kits (R&D Systems, Shanghai, China).

### Detection of cerebral edema

Five rats were taken from each group. The brain tissue was removed after anesthesia, and the wet weight was weighed. Then, the tissue blocks were finely cut and baked in an electric oven at 105°C for 48 hours to constant weight (dry weight). The brain edema was assessed according to the following formula: Tissue moisture content = (wet weight-dry weight)/wet weight × 100% (Accuracy of 0.2 mg).

### Hematoxylin & eosin (H&E) staining

Frozen brain tissue sections were rewarmed, hydrated, stained with hematoxylin stain, fractionated with 1% hydrochloric acid ethanol and flushed with tap water. After staining with eosin stain, the sections were dehydrated with an alcohol gradient, transparentized with xylene, and mounted with sealing resin. The pathological alterations of brain tissues were reviewed under a microscope.

### Immunohistochemistry (IHC)

After routine paraffin-embedding and sectioning (4 μM) of brain tissues, xylene dewaxing and gradient alcohol hydration were performed. With the endogenous peroxidase being inactivated by 3% H_2_O_2_ for 10 min, the microwave repair was administered with 0.01 mol/L sodium citrate buffer (pH = 6.0, 15 min). After being blocked for 20 min with 5% bovine serum albumin (BSA), the sections were incubated with the primary Anti-Caspase-3 antibody (1:100, ab13847), Anti-AMPK antibody (1:200: ab218040), and Anti-Iba-1 antibody (1:200, ab178846) at 4°C overnight. The next day, the secondary Anti-Rabbit (1:2500, ab6721) was added and maintained at RT for 20 min. The sections were washed with PBS and then colored with DAB. After hematoxylin re-staining, the sections were subjected to dehydration, transparency, and blocking for microscope examination. The above antibodies were from Abcam (MA, USA).

Judgment criteria for immunohistochemical results: five fields of view were randomly chosen per section at 400 magnification. Positive cells were indicated by brownish-yellow nuclei or cytoplasm. One hundred cells were counted in each field of view and scored according to the number of positive cells as a percentage of the total number of cells (a): A percentage of less than 5% was scored 0, a percentage between 5% and 25% was scored 1, a percentage between 26% and 50% was scored 2, and a percentage greater than 50% was scored 3. Positive intensity (b): Cells that were uncolored or stained a pale yellow in line with the background were scored as 0, light tan as 1, tan as 2 and brown as 3. The scores were based on the two indicators (points = a × b), with 2-3 scores being weakly positive (+), 4–6 scores being positive (++), and 7–9 scores being strongly positive (++++). The mean number of positive and strongly positive cells in each group was observed and compared.

### Terminal deoxynucleotidyl transferase-mediated dUTP-biotin nick end labeling (TUNEL) staining

The paraffin sections of the brain were collected and processed according to the TUNEL Apoptotic Detection Kit instructions. Three sections were taken from each specimen, and five non-overlapping high-power (×400) fields of the surrounding cortex were randomized from each section. The number of TUNEL-positive cells, i.e., apoptotic cells and the total cell number, were calculated using the Image-Pro Plus image analysis software. The apoptotic index (AI) = apoptotic cell number/total cell number × 100%.

### Quantitative real-time PCR (qRT-PCR)

Total RNA was isolated from each group of cells and tissues by applying the TRIzol reagent (Invitrogen, Carlsbad, CA, USA). Reverse transcription was implemented using the PrimeScript™ RT Reagent kit (Invitrogen, Shanghai, China) as per the manufacturer’s directions to obtain the first strand of cDNA. Then, the strand served as a template for qPCR using the Bio-Rad CFX96 quantitative PCR system and SYBR. PCR was performed with pre-denaturation at 95°C for 5 min, denaturation at 95°C for 15 s, and annealing at 60°C for 30 s. β-actin served as a housekeeping gene for AMPK. The target gene expression = 2^−ΔΔCT^. The above cell experiment was repeated three times, and the animal experiment was conducted five times. Primer sequences are as follows:

**Table:** 

**Gene**	**Primer sequence**
AMPK	F: CGGCTTTCCTTTTCGTCCAA R: CTCAACCGGCAGAAGATTCG
β-Actin	F: CCTGCTTGCTGATCCACATC R: CCTCTATGCCAACACAGTGC

### Statistical analysis

The experimental data in this study were processed with SPSS18.0 statistical software (SPSS Inc., Chicago, IL, USA) and GraphPad Prism 8 (GraphPad Software, USA). Data were expressed as mean ± standard deviation (x ± s). *t* test was adopted to compare the difference of the mean between the two groups, and a one-way analysis of variance was employed to compare the difference between the data of multiple groups (>2). *P < 0.05* was considered statistically significant.

### Ethics statement

Our study was approved by the Animal Ethics Committee of Shanxi Provincial People’s Hospital.

### Data availability statement

The data sets used and analyzed during the current study are available from the corresponding author on reasonable request.

## RESULTS

### Mat alleviated LPS-mediated neuronal damage

**Figure 1:**
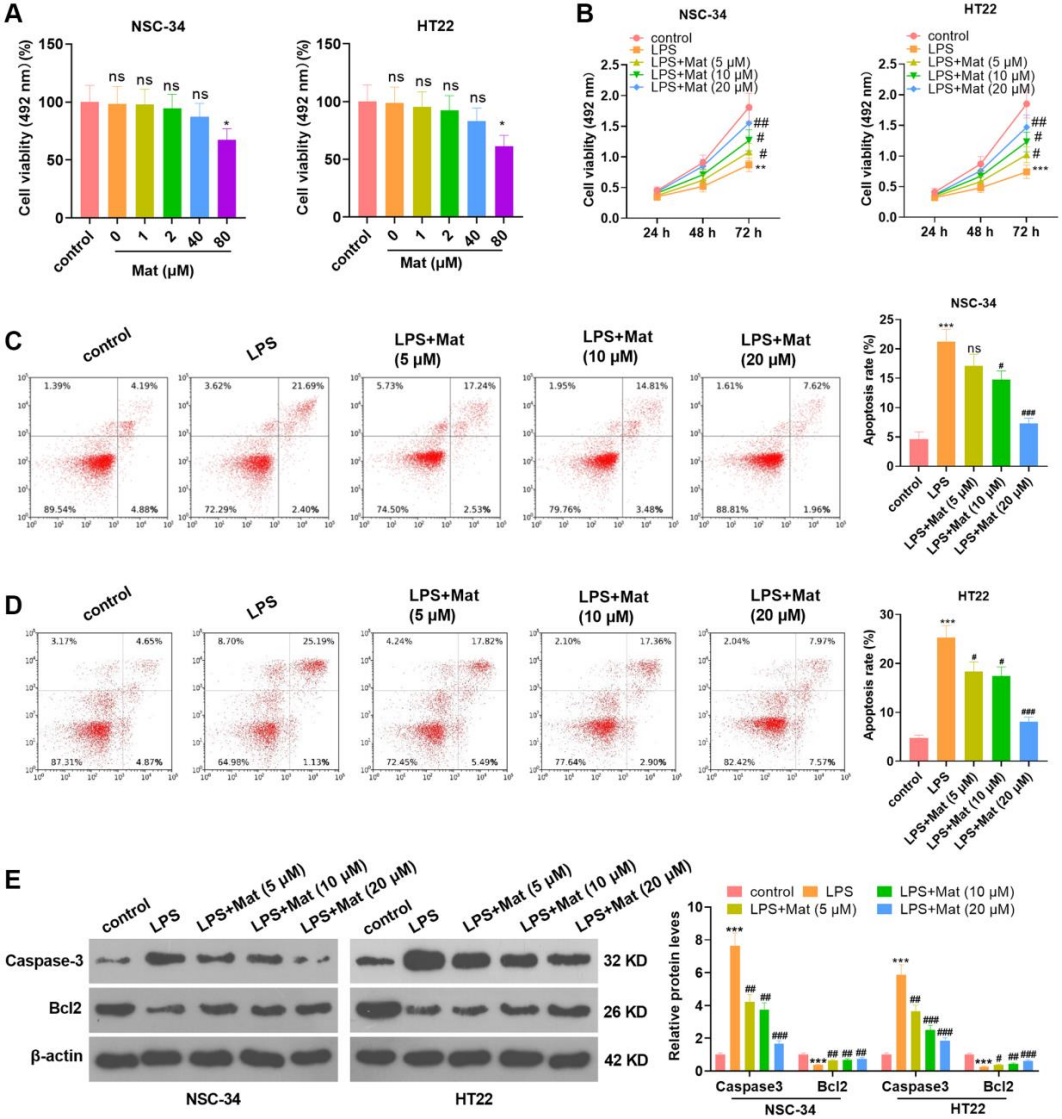
**Mat attenuated the LPS-mediated neuronal damage.** Different concentrations (0 to 80 μg/mL) of Mat were taken to treat NSC-34 and HT22 neurons for 48 hours. (**A**) The toxicity of Mat on neurons was gauged by MTS analysis. On the basis of 1 μg/mL LPS treatment for 24 hours, different concentrations (5 to 20 μg/mL) of Mat were taken to treat NSC-34 and HT22 cells for 48 hours. (**B**) Cell proliferation was assessed by MTS analysis at the 24th, 48th and 72nd hours after Mat treatment. (**C**–**D**) Apoptosis was examined by FCM. (**E**) WB verified the expression of Caspase3 and Bcl2. ^*^P < 0.05, ^***^P < 0.001 (vs. control group). ^+^*P* < 0.05, ^++^*P* < 0.01, ^++^*P* < 0.001 (vs. LPS group).

With the purpose of exploring the effect of Mat on neurons, different concentrations of Mat (0, 1, 2, 40, 80 μM) were applied to NSC-34 and HT22 neurons. MTS analysis results illustrated that Mat notably hindered cell viability when its concentration was greater than 40 μM (*P* < 0.05, Figure 1A), suggesting that the toxicity of Mat to neurons was minimal. Then, NSC-34 and HT22 cells were treated with varying concentrations of Mat (5, 10, 20 μM) following LPS treatment. MTS analysis uncovered that LPS distinctly impeded cell proliferation (vs. the control group). However, Mat (>5 μM) heightened the proliferative ability of cells (vs. the LPS alone intervention group) (*P* < 0.05, Figure 1B). As testified by FCM data, LPS heightened apoptosis (vs. the control group), while Mat (>5 μM) significantly repressed apoptosis in contrast to that of the LPS group (*P* < 0.05, Figure 1C–1D). WB data displayed that LPS elevated the expression of pro-apoptotic protein Caspase3 and dampened the profile of anti-apoptotic protein Bcl2 (vs. the control group). However, Mat attenuated this effect (vs. the LPS group) (*P* < 0.05, Figure 1E). These outcomes illustrated that Mat weakened the anti-proliferative and pro-apoptotic effects of LPS on neurons, thus mitigating the damage of LPS on neurons.

### Mat attenuated LPS-mediated oxidative stress and inflammation in microglia

**Figure 2:**
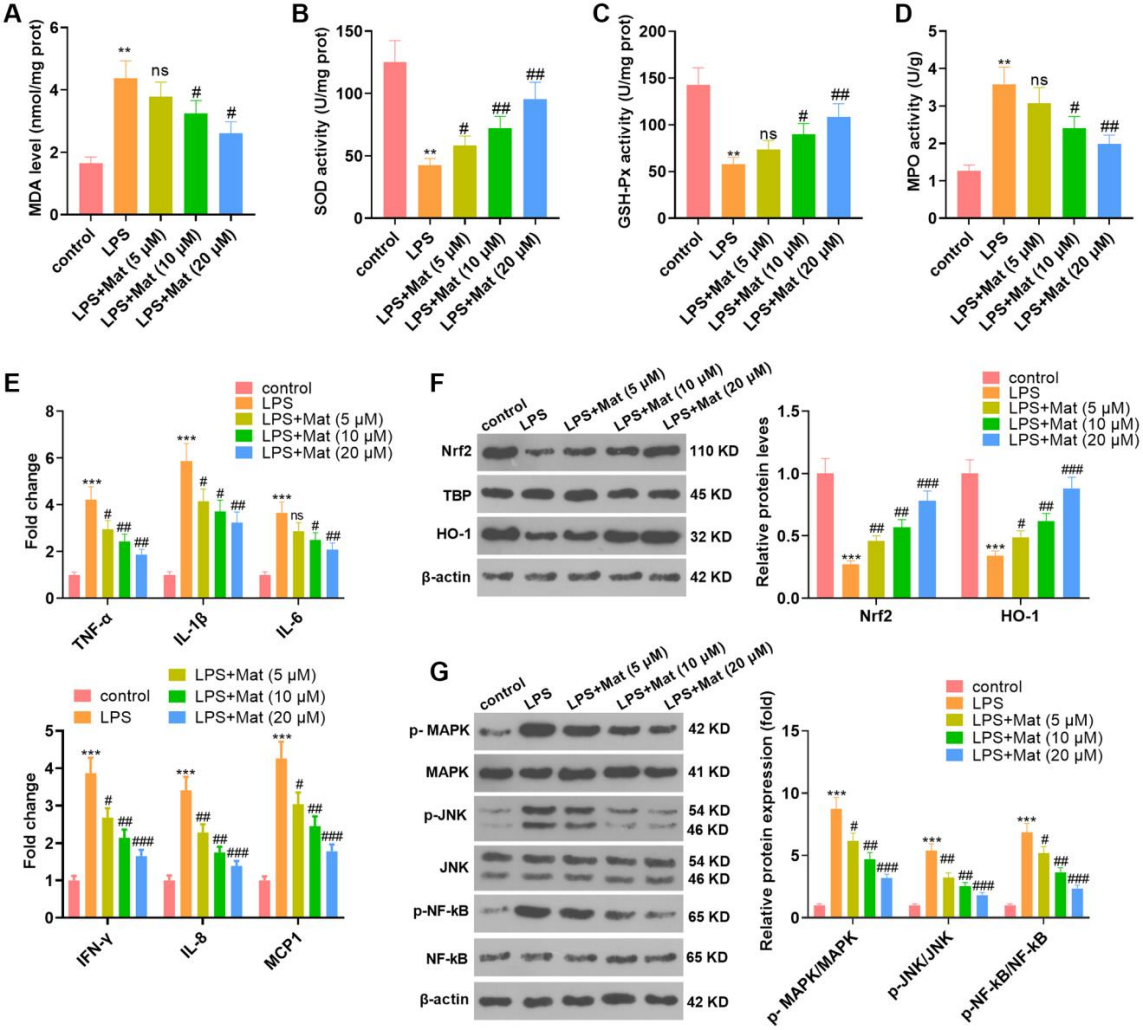
**Mat mitigated LPS-mediated oxidative stress and inflammation in microglia.** After 1 μg/mL LPS treatment for 24 hours, NSC-34 and HT22 cells were treated with varying concentrations (5 to 20 μg/mL) of Mat for 48 hours. (**A**–**D**) MDA production and SOD, CAT and GSH-Px activities were measured by utilizing the BCA protein assay kits. (**E**) ELISA was implemented to test the cellular levels of TNF-α, IL-1β, IL-6, IFN-γ, IL-8 and MCP1. (**F**–**G**) The profiles of Nrf2, HO-1, MAPK P38, JNK and NF-κB were compared by WB. ^**^P < 0.01, ^***^P < 0.001 (vs. control group). ^#^*P* < 0.05, ^##^*P* < 0.01, ^##^*P* < 0.001 (vs. LPS group).

To clarify the effect of Mat on microglia, BV2 were treated with different concentrations (5 to 20 μM) of Mat following LPS treatment. The results of the BCA protein detection kit manifested that by contrast with the control group, LPS enhanced the production of MDA and hampered the activities of SOD, CAT and GSH-Px, while Mat (>5 μM) significantly impeded this effect (vs. the LPS group) (*P* < 0.05, Figure 2A–2D). ELISA results hinted that LPS heightened the generation of inflammatory cytokines (TNF-α, IL-1β, IL-6, IFN-γ, IL-8 and MCP1), while Mat repressed the production of these inflammatory cytokines compared with that of the LPS group (*P* < 0.05, Figure 2E). The profiles of oxidative stress proteins (Nrf2 and HO-1) and inflammatory proteins (MAPK, JNK and NF-κB) were monitored by WB. As a result, in contrast to the control group, LPS repressed the profiles of Nrf2 and HO-1 and strengthened the phosphorylation of MAPK, JNK and NF-κB. Nevertheless, further application of Mat following LPS treatment brought about the reverse effect (*P* < 0.05, Figure 2F–2G). Overall, LPS induced oxidative stress and inflammation in microglia, and Mat attenuated the effect of LPS.

### Mat eased CLP-induced acute brain injury in rats

**Figure 3:**
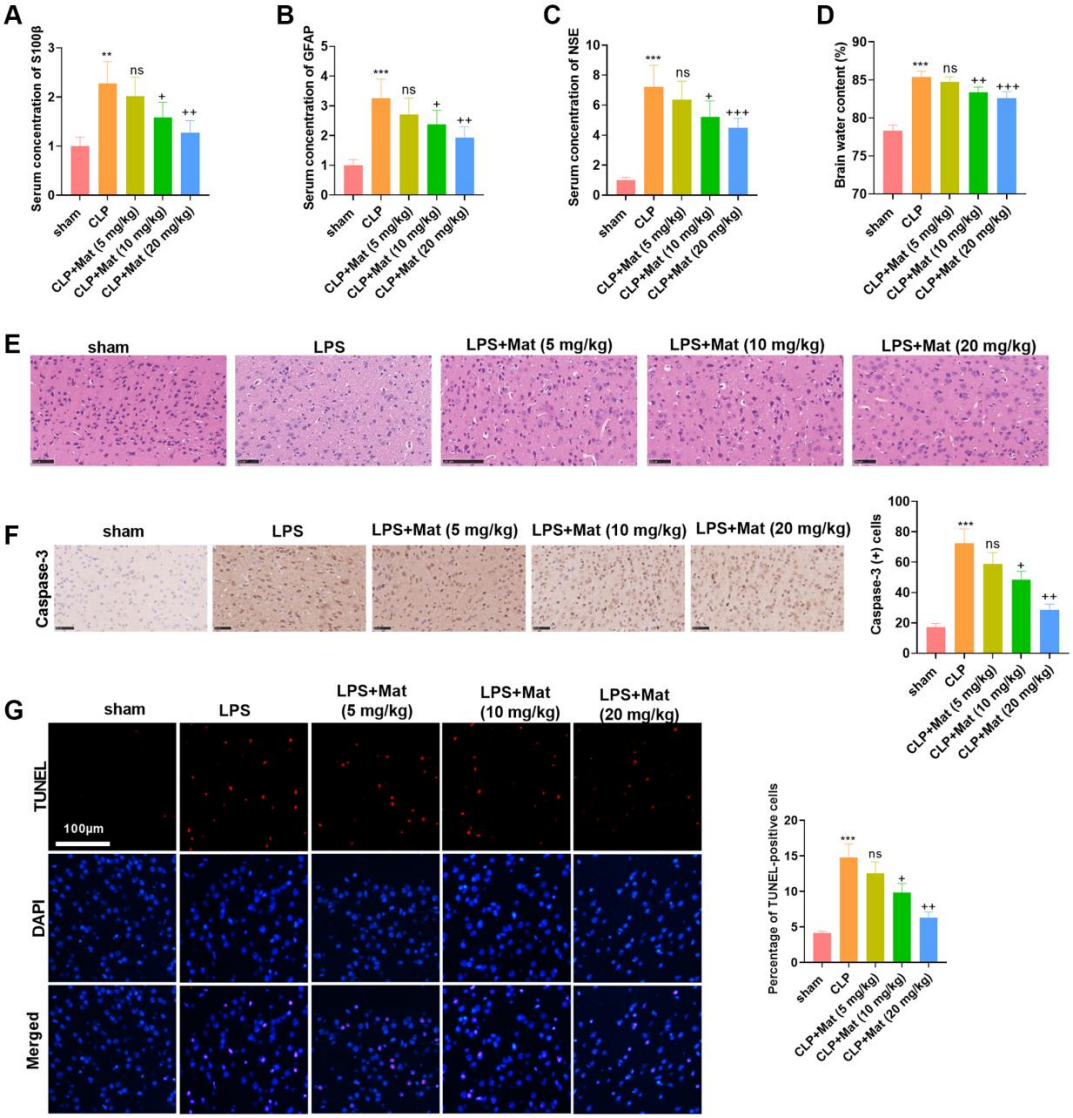
**Mat alleviated CLP-induced acute brain injury in rats.** A rat sepsis model was constructed using the CLP surgery, based on which rats were given oral doses of Mat at different concentrations (5 to 20 mg/kg) for 48 hours. (**A**–**C**) Blood analysis was utilized to compare the concentrations of S100β, GFAP and NSE in the rat serum. (**D**) Brain edema was measured using wet-dry method. (**E**) Brain tissues were stained with H&E. (**F**) IHC was taken to calculate the Caspase-3-positive cell number. (**G**) TUNEL staining was performed to detect tissue apoptosis. ^**^P < 0.01, ^***^P < 0.001 (vs. sham group). ns *P* > 0.05, ^+^*P* < 0.05, ^++^*P* < 0.01, ^++^*P* < 0.001 (vs. CLP group).

A rat sepsis model was constructed by CLP surgery, based on which rats were given oral doses of Mat at different concentrations (5 to 20 mg/kg). Blood analysis revealed that serum concentrations of S100β, GFAP, and neuron-specific enolase (NSE) were significantly increased after CLP, while Mat (>5 mg/kg) dampened the production of S100β, GFAP, and NSE (vs. the CLP group) (*P* < 0.05, Figure 3A–3C). No significant effect of CLP and Mat on brain edema was observed in the wet/dry method (*P* > 0.05, Figure 3D). H&E staining of rat brain tissues exhibited that the neurons in the sham group were basically structurally intact with normal neuronal morphology, clear cytoplasm, and uniformly clear cortical nuclei, while those in the CLP group were abnormally disorganized with vacuoles. However, the use of Mat on the basis of CLP attenuated the damage of CLP to brain tissues and neurons (Figure 3E). IHC results testified that CLP elevated the Caspase-3 expression in the cortex, while Mat (>5 mg/kg) significantly dampened the Caspase-3 profile (vs. the CLP group) (*P* < 0.05, Figure 3F). Then, TUNEL staining data revealed that CLP heightened the number of apoptotic cells, while Mat (>5 mg/kg) repressed cell apoptosis in comparison to that of the CLP group (*P* < 0.05, Figure 3G). These outcomes manifested that Mat had an inhibitory effect on CLP-induced acute brain injury in rats.

### Mat lightened CLP-mediated inflammation and oxidative stress

**Figure 4:**
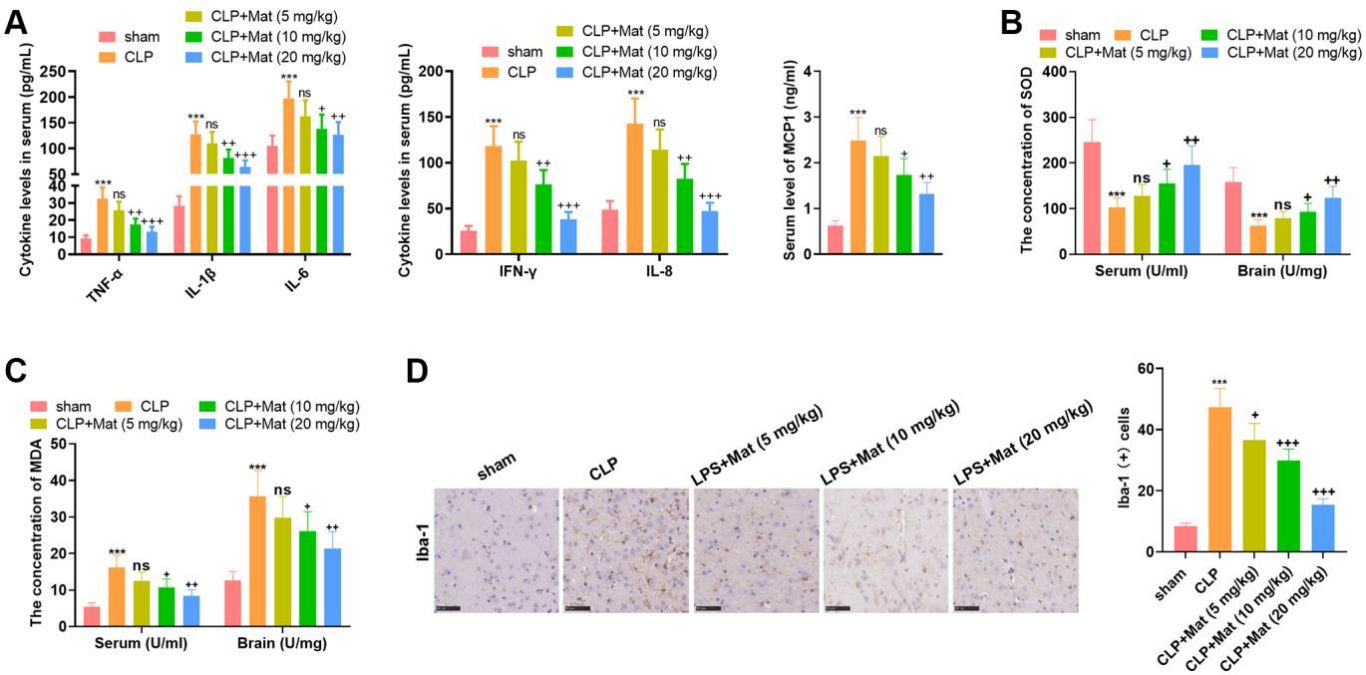
**Mat repressed CLP-mediated inflammation and oxidative stress.** Experimental animals were treated like that in Figure 3. (**A**) ELISA was adopted to determine the cellular contents of TNF-α, IL-1β, IL-6, IFN-γ, IL-8 and MCP1. (**B**–**C**) The BCA protein assay kits were employed to test the concentrations of MDA and SOD in the serum and brain tissue. (**D**) The Iba-1 positive cell number was checked by IHC. ^***^P < 0.001 (vs. sham group). ns *P* > 0.05, ^+^*P* < 0.05, ^++^*P* < 0.01, ^++^*P* < 0.001 (vs. CLP group).

The treatment of experimental animals is shown in Figure 3. ELISA results confirmed that LPS facilitated the generation of TNF-α, IL-1β, IL-6, IFN-γ, IL-8 and MCP1, while Mat (>5 mg/kg) treatment following CLP administration curbed the production of TNF-α, IL-1β, IL-6, IFN-γ, IL-8 and MCP1 (*P* < 0.05, Figure 4A). The BCA protein detection kit was adopted to test the concentration of oxidative stress molecules in the serum and brain tissues. It turned out that CLP facilitated the MDA production and hampered the SOD activity (vs. the sham group), while Mat declined the MDA level and increased the SOD activity (vs. the CLP group) (*P* < 0.05, Figure 4B–4C). After the CLP surgery, IHC results showed a rapid increase in the Iba-1 positive cell number, which was suppressed by Mat (vs. the CLP group) (*P* < 0.05, Figure 4D). These data revealed that Mat hampered CLP-mediated microglial activation. Thus, Mat inhibited CLP-mediated oxidative stress, inflammation, and microglial activation *in vivo*.

### Mat abated the CLP-activated MAPK and NF-κB axis

**Figure 5:**
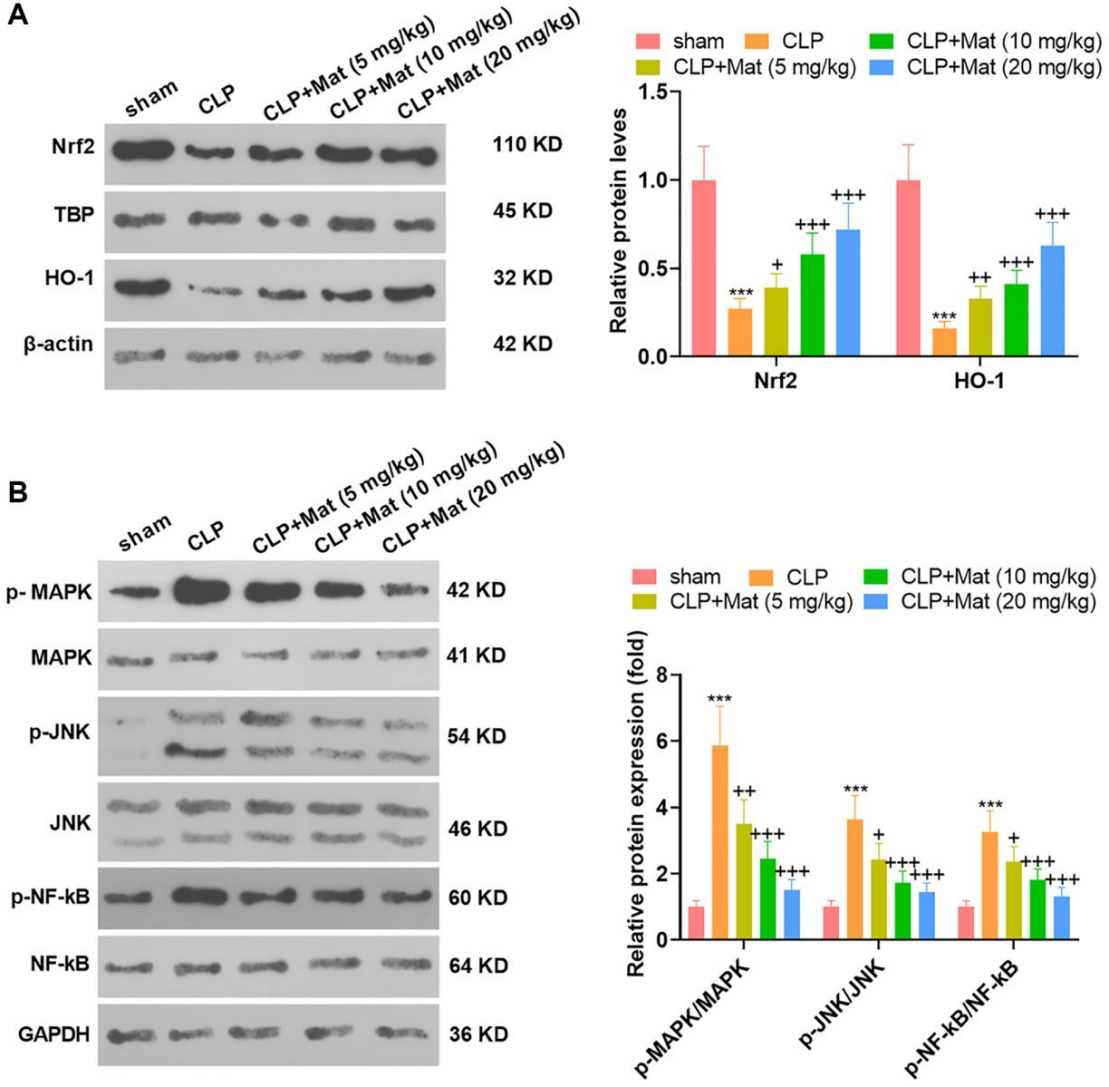
**Mat choked the CLP-activated MAPK and NF-κB pathways.** Experimental animals were treated like that in Figure 3. (**A**–**B**) The profiles of Nrf2, HO-1, MAPK P38, JNK and NF-κB were tested by WB. ^***^P < 0.001 (vs. sham group). ns *P* > 0.05, ^+^*P* < 0.05, ^++^*P* < 0.01, ^++^*P* < 0.001 (vs. CLP group).

The treatment of experimental animals was the same as that in Figure 3. After the CLP surgery, the expression of antioxidant proteins Nrf2 and HO-1 was suppressed, while the phosphorylation of inflammatory proteins MAPK, JNK and NF-κB was promoted, as evidenced by WB. Nevertheless, Mat treatment at varying concentrations following the CLP surgery significantly suppressed the inhibition of CLP on Nrf2 and HO-1 and its facilitation on MAPK, JNK and NF-κB (*P* < 0.05, Figure 5A–5B). These outcomes suggested that Mat repressed the CLP-activated MAPK and NF-κB pathways.

### Mat heightened the AMPK expression

**Figure 6:**
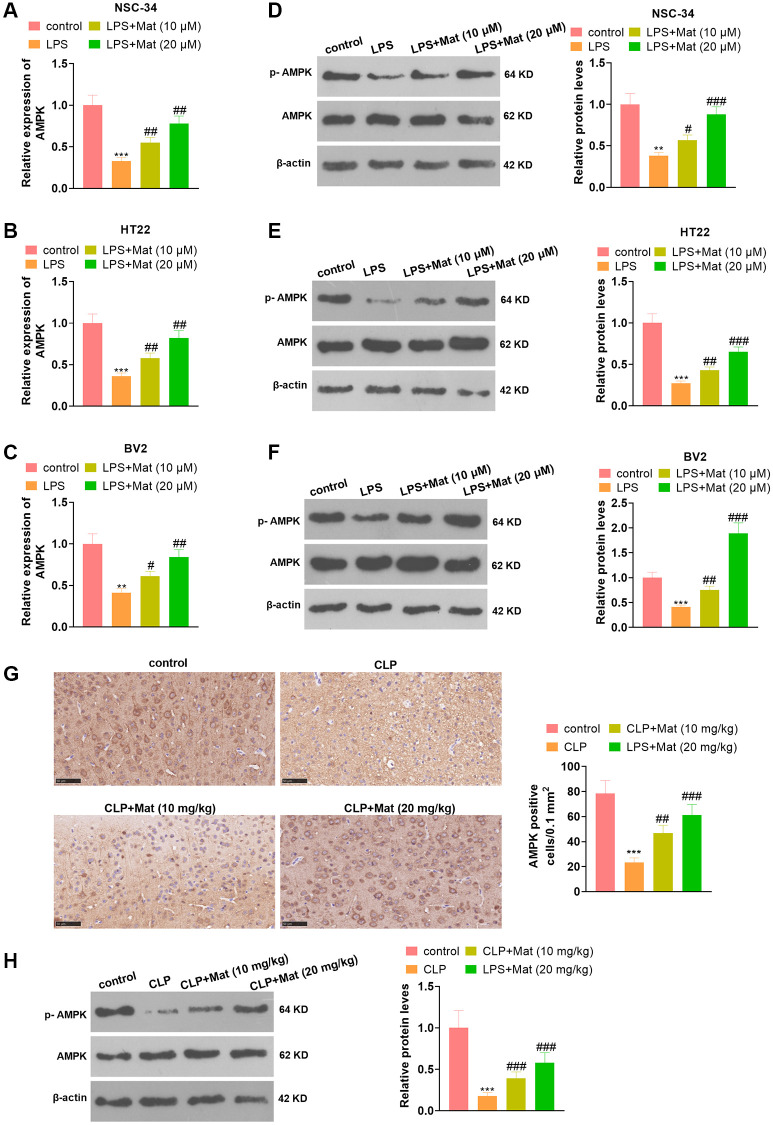
**Mat facilitated the AMPK expression.** In addition to LPS (1 μg/mL) treatment for 24 hours, NSC-34, HT22 and BV2 cells were intervened with Mat (10, 20 μM) for 48 hours. (**A**–**C**) The AMPK expression at the mRNA level was examined by qRT-PCR. (**D**–**F**) The AMPK protein profile was verified by WB. Rats were orally administered with Mat (10, 20 mg/kg) following CLP treatment. (**G**) The AMPK expression in tissues was monitored by IHC. (**H**) The AMPK phosphorylation was measured by WB. ^**^P < 0.01, ^***^P < 0.001 (vs. control group). ^#^*P* < 0.05, ^##^*P* < 0.01, ^##^*P* < 0.001 (vs. LPS group).

NSC-34, HT22 and BV2 cells were processed with LPS alone or in combination with Mat (10, 20 μM). qRT-PCR and WB affirmed that LPS repressed the AMPK expression at mRNA and protein levels (vs. the control group). In contrast, compared with the LPS group, Mat elevated the AMPK expression concentration-dependently (*P* < 0.05, Figure 6A–6F). Besides, Mat (10, 20 mg/kg) and CLP were applied jointly to treat the rats. IHC results illustrated that CLP abated the AMPK expression in tissues (vs. the control group), while Mat strengthened the AMPK level (vs. the CLP group) (*P* < 0.05, Figure 6G). WB hinted that the AMPK phosphorylation was reduced by CLP, while it was increased after Mat administration (vs. the CLP group) (*P* < 0.05, Figure 6H). These results manifested that Mat elevated the AMPK expression.

### Inhibiting AMPK weakened the Mat-mediated neuronal protection and anti-inflammatory effects

**Figure 7:**
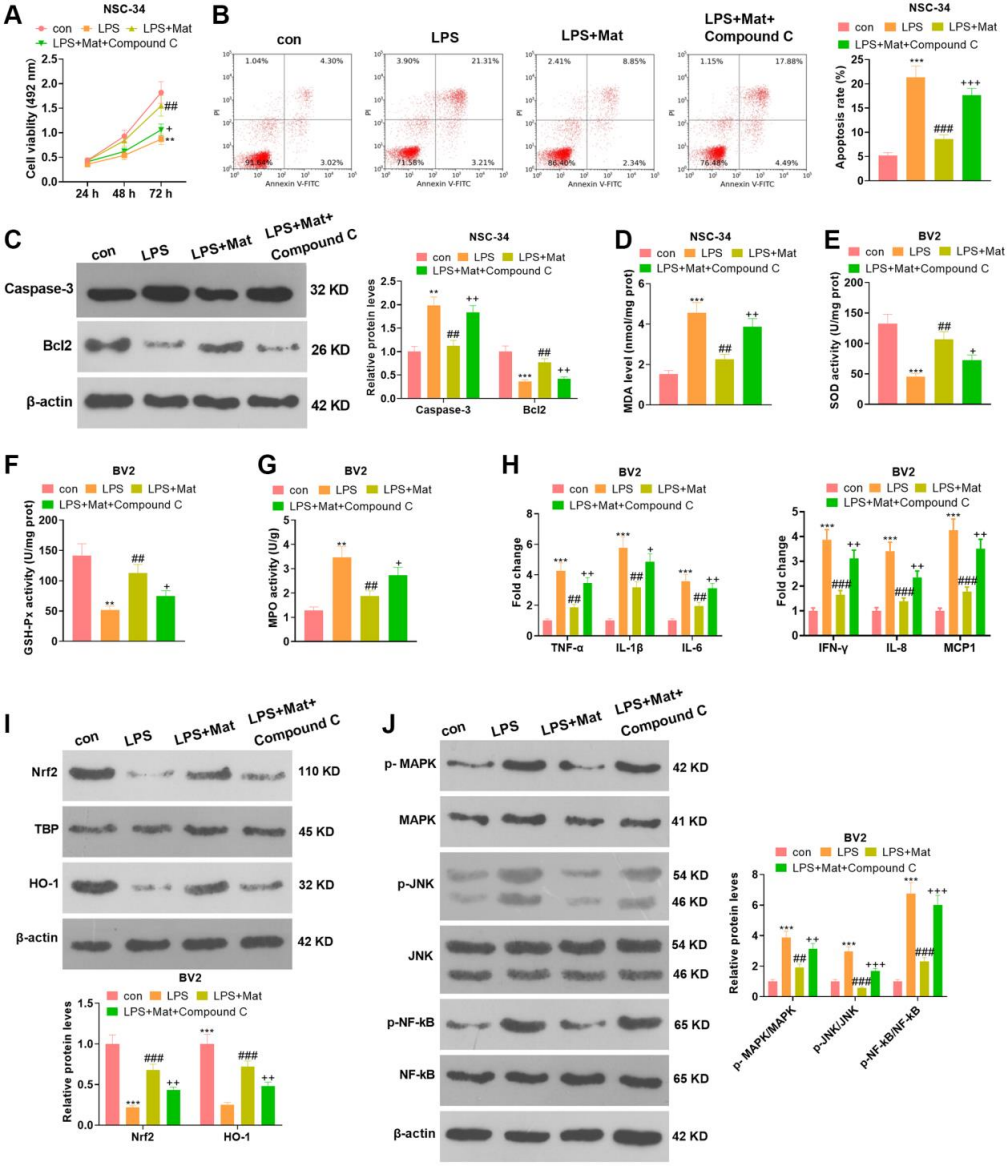
**Attenuating AMPK abated Mat-mediated neuron-protective and anti-inflammatory effects.** NSC-34 and BV2 cells were treated with Mat (20 μM) alone or in combination with Compound C for 48 hours following LPS (1 μg/mL) treatment for 24 hours. (**A**–**B**) proliferation and apoptosis of NSC-34 cells were measured by CCK8. (**C**) The expression of Caspase-3 and Bcl2 was tested by WB. (**D**–**G**) Detection of MDA, SOD, CAT and GSH-Px levels in NSC-34 was performed using the BCA protein assay kit. (**H**) ELISA was applied for gauging the production of TNF-α, IL-1β, IL-6, IFN-γ, IL-8 and MCP1 in NSC-34. (**I**–**J**) The expression of MAPK and JNK, as well as NF-κB in BV2 microglia, was compared by WB. ^**^P < 0.01, ^***^P < 0.001 (vs. control group). ^##^*P* < 0.01, ^##^*P* < 0.001 (vs. LPS group). ^+^*P* < 0.05, ^++^*P* < 0.01 (vs. LPS+Mat group).

NSC-34 and BV2 cells were treated with Mat (20 μM) alone or in combination with Compound C (the AMPK pathway inhibitor) following LPS treatment. Cell counting kit-8 (CCK-8) assay disclosed that Compound C signally abated NSC-34 cell proliferation (vs. the LPS+Mat group) (*P* < 0.05, Figure 7A). FCM revealed that the apoptotic rate of NSC-34 was increased after Compound C treatment (vs. the LPS+ Mat group) (*P* < 0.05, Figure 7B). The expression of apoptosis-related proteins was compared by WB. As a result, Compound C significantly facilitated Caspase-3 and curbed Bcl2 expression (vs. the LPS+Mat group) (*P* < 0.05, Figure 7C). The results of the BCA protein assay kit exhibited significant facilitation in MDA release and notable attenuation in SOD, CAT and GSH-Px activities after Compound C treatment (vs. the LPS+Mat group) (*P* < 0.05, Figure 7D–7G)). Additionally, ELISA testified that Compound C significantly facilitated the production of TNF-α, IL-1β, IL-6, IFN-γ, IL-8 and MCP1 in NSC-34 (vs. the LPS+Mat group) (*P* < 0.05, Figure 7H). Moreover, WB indicated that Compound C hampered the expression of Nrf2 and HO-1 and augmented the phosphorylation of MAPK, JNK and NF-κB in BV2 cells (vs. the LPS+ Mat group) (*P* < 0.05, Figure 7I–7J). Hence, attenuating AMPK impeded Mat-mediated neuron-protective and anti-inflammatory effects.

## DISCUSSION

Brain injury is often observed after sepsis, and it is implicated in the direct damage (such as cerebral edema, ischemia, epilepsy) or secondary/indirect injuries (such as hypotension, hypoxemia, hypocapnia, and hyperglycemia) of sepsis to the brain [24]. According to previous studies, inducing sepsis using LPS [25–26] or CLP [27–28] has been widely recognized and applied. Therefore, this study was conducted based on the construction of the LPS-induced cellular sepsis model and CLP-mediated rat sepsis model. We found that Mat suppressed LPS/CLP-mediated damage, inflammation and oxidative stress. Besides, Mat showed a facilitative effect on AMPK and an inhibitory effect on the MAPK and NF-κB pathways *in vivo*.

Lignans have a diverse structure consisting of two phenyl propane units with different degrees of oxidation in the propane moiety and different substitution patterns in the aromatic rings [29]. Multiple Lignans have anti-sepsis effects. For example, Sesamint restrains intestinal injury in septic mice by down-regulating the HMGB-1/TLR4/IL-33 pathway [30]. Schisandrin B attenuates LPS-induced sepsis in mice by down-regulating TLR4 via miR-17-5p [31]. Nordihydroguaiaretic acid curbs sepsis-induced lung injury in rats [32]. Mat is one of the natural lignans found in plants and has anti-inflammatory and antioxidant activities [33–34]. Here, MTS analysis was employed to probe the toxicity of Mat (1 to 80 μg/mL) to neurons before exploring the effect of Mat on neurons. As a result, only a high concentration (80 μg/mL) of Mat showed a significant cytotoxic effect. Based on this, we applied 5, 10, and 20 μg/mL of Mat for subsequent experiments. Notably, Mat dampens LPS-induced BV2 inflammation and migration through the activation of NF-κB and regulation of the Src axis [35]. Similarly, we discovered that Mat abated the anti-proliferative and pro-apoptotic effects of LPS on neurons and alleviated LPS-mediated microglial oxidative stress and inflammation.

After cellular experiments, we constructed a rat sepsis model using LPS to further clarify the role of Mat. Several studies have demonstrated that S100β, GFAP and NSE are serum markers of sepsis-associated brain diseases [36–37]. In the present article, we discovered that Mat repressed the promotion of LPS on them. Further experiments revealed that although neither LPS nor Mat showed significant effects on brain edema, Mat ameliorated the pathological changes of LPS on rat brain tissues and hindered LPS-mediated neuronal apoptosis, which suggested that Mat alleviated CLP-induced acute brain injury in rats. On the other hand, Honokiol, a natural polyphenol from traditional Chinese herbal medicine magnolia, can reduce sepsis-induced organ damage, such as kidney injury [38–39] and lung injury [40], through its anti-inflammatory and antioxidant effects. In parallel, we discovered that Mat attenuated CLP-mediated inflammation and oxidative stress by restraining the production of TNF-α, IL-1β, and IL-6, strengthening the SOD activity, and abrogating MDA levels in rats’ brain tissues.

Multiple studies have stated that the MAPK and NF-κB pathways regulate microglial inflammation. Oxymatrine exhibits anti-neuroinflammatory effects on Aβ1-42-induced primary microglia through inhibition of NF-κB and MAPK [41]. In addition, sulforaphane extracted from Cruciferae vegetables attenuates microglia-mediated neuronal necrosis by down-regulating the MAPK /NF-κB pathway in LPS-activated BV-2 microglia [42]. Here, we found that Mat reduced the expression of CLP-mediated oxidative stress proteins (Nrf2 and HO-1) and inflammatory proteins (MAPK, JNK and NF-κB). The regulatory role of AMPK in neurological diseases has also been demonstrated. For example, Safflower yellow B protects the rat brain from cerebral ischemia-reperfusion injury through the AMPK/NF-κB pathway [43]. Other studies have manifested that AMPK signaling activation has the potential to protect against Parkinson’s disease [44]. Here, we found that Mat weakened the inhibitory effect of LPS/CLP on the AMPK pathway, and the AMPK inhibitor repressed Mat’s protective effect on neurons and its anti-inflammatory and antioxidant effects on microglia. Moreover, inhibition of AMPK strengthened phosphorylation of MAPK, JNK and NF-κB, which further suggested that Mat exerted an anti-inflammatory and antioxidant role through the AMPK-mediated MAPK and NF-κB pathways, thereby alleviating sepsis. However, this study is still limited to the cellular and animal experiments, and the exact dose and method of Mat application, as well as its side effects, are not yet known. Other molecular mechanisms by which Mat plays its anti-inflammatory and antioxidant effects need further investigation.

Overall, the potential value of Mat has been found in this study. Mat hampered LPS-induced neuronal damage and CLP-induced acute brain injury in mice. Additionally, Mat choked inflammation and oxidative stress in microglia and mice. Mechanistic studies have substantiated that Mat attenuates sepsis-mediated brain damage by inactivating the MAPK and NF-κB pathways. In a word, this research reveals the inhibitory effect of Mat on sepsis-induced brain injury, which provides a theoretical basis and research strategy for clinical intervention in treating sepsis-induced brain injury.
